# In silico drug repositioning based on the integration of chemical, genomic and pharmacological spaces

**DOI:** 10.1186/s12859-021-03988-x

**Published:** 2021-02-08

**Authors:** Hailin Chen, Zuping Zhang, Jingpu Zhang

**Affiliations:** 1 School of Software, East China Jiaotong University, Nanchang, 330013 China; 2 School of Computer Science and Engineering, Central South University, Changsha, 410083 China; 3 School of Computer and Data Science, Henan University of Urban Construction, Pingdingshan, 467000 China

**Keywords:** Drug repositioning, Drug feature, Similarity fusion

## Abstract

**Background:**

Drug repositioning refers to the identification of new indications for existing drugs. Drug-based inference methods for drug repositioning apply some unique features of drugs for new indication prediction. Complementary information is provided by these different features. It is therefore necessary to integrate these features for more accurate in silico drug repositioning.

**Results:**

In this study, we collect 3 different types of drug features (i.e., chemical, genomic and pharmacological spaces) from public databases. Similarities between drugs are separately calculated based on each of the features. We further develop a fusion method to combine the 3 similarity measurements. We test the inference abilities of the 4 similarity datasets in drug repositioning under the guilt-by-association principle. Leave-one-out cross-validations show the integrated similarity measurement *IntegratedSim* receives the best prediction performance, with the highest AUC value of 0.8451 and the highest AUPR value of 0.2201. Case studies demonstrate *IntegratedSim* produces the largest numbers of confirmed predictions in most cases. Moreover, we compare our integration method with 3 other similarity-fusion methods using the datasets in our study. Cross-validation results suggest our method improves the prediction accuracy in terms of AUC and AUPR values.

**Conclusions:**

Our study suggests that the 3 drug features used in our manuscript are valuable information for drug repositioning. The comparative results indicate that integration of the 3 drug features would improve drug-disease association prediction. Our study provides a strategy for the fusion of different drug features for in silico drug repositioning.

## Background

Despite continuous advances in modern technologies, the process of traditional drug discovery is still extremely time-consuming and costly. According to a recent study [1], it takes over 10 years and more than $2 billion to bring a new drug to market. Moreover, the risk of failure during drug discovery is significantly high. Most drug leads could not pass beyond the early stage of development because of toxicity, and lack of efficacy or adverse side-effects could further prevent testing drugs from entering clinical trials. Therefore, improving research and development (R&D) productivity becomes the most important priority for the global pharmaceutical industry [2].

Drug repositioning [3], which aims to find new indications for approved or investigational drugs, has emerged as an important alternative to the traditional drug discovery. As it uses de-risked drug compounds, drug repositioning has the potential to reduce development time and increase success ratio compared to developing an entirely new drug for disease treatment [4]. Some successful examples of drug repositioning have been reported. A well-known instance is sildenafil, which has been repurposed from an antihypertensive drug to the treatment of erectile dysfunction. Existing antivirals, such as baloxavir, azvudine and darunavir, are repurposed to fight the current COVID-19 pandemic [5].

With the accumulation of biomedical data, computational approaches exploiting multi-source information for drug repositioning have been continuously proposed [6–27]. These methods can be roughly categorized as drug-based and disease-based (see Review [28] for more details). Drug-based approaches are preferred when rich chemical or pharmacological data for drugs are available. For example, under the principle that drugs with chemical similarities could suggest shared biological activity, Keiser et al. [7] applied a similarity ensemble approach (SEA) to evaluate the 2D structural similarity of drugs to identify new drug–target interactions for drug repositioning. Based on the hypothesis that the mechanism of actions (MoA) of two drugs would be same if they induced the same side effects, Yang and Agarwal [8] used clinical side-effects of drugs as features to build Naive Bayes models to predict indications for diseases. Because protease is a common target for SARS-CoV-2, HIV-1 and hepatitis C viral (HCV) strains. FDA approved HIV-1 protease inhibitors and HCV protease inhibitors have been screened to be potential effective drugs against the COVID-19 [27]. Considering the fact that a drug usually acts on multiple targets, Rutherford et al. [14] extracted drug-disease associations for drug repositioning using the interactions between disease-related genes and drug targets. For these methods, different drug features are applied to address the drug repositioning problem from different angles.

Generally, these drug-based approaches compare some unique signature of a drug against that of another one. The signature of a drug could be mainly derived from three categories of data: chemical structures, genomic data and adverse event profiles. As we know, collection bias and noise may exist in these data and some are even not complete. Meanwhile, complementary information exists in these different types of data. Therefore, it is necessary to combine these data for a comprehensive understanding of drug’s MoA. However, integrating these different kinds of data to improve in silico drug repositioning is an open question till now.

In this paper, we first collect 3 types of drug data (i.e., drug substructures, drug targets and drug side-effects) from public databases. Drug–drug similarities are then calculated based on each of the three types of features. A method using propagation to integrate the three similarity measurements is proposed. Under the guilt-by-association principle, we finally test their ability to infer drug-disease associations for drug repositioning. Experimental results based on cross-validations and case studies show that the integrated similarity measurement outperforms each of the 3 similarity measurements. We also compare our fusion method with 3 state-of-the-art similarity-integration methods and our method shows superior prediction performance in drug repositioning.

## Results

### Evaluation metrics

In order to evaluate the prediction performance of the 4 similarity measurements, we implement leave-one-out cross-validations (LOOCVs) on the 548 drugs. For each drug, we consider it as a new one and leave it out once as the testing data. We remove all the associated diseases of the testing drug from our dataset. The remaining 547 drugs with indication information and similarity measurement are taken as the training data.

For each drug, we prioritize the whole candidate diseases according to the scores derived from Eq. (8) (see “Methods”). When the score of a predicted association exceeds a given threshold, we consider it as a positive prediction; otherwise, a negative prediction. True positive rate (TPR), false positive rate (FPR), Precision (P) and Recall (R) are calculated by varying the thresholds to plot ROC and PR curves. Area under ROC curve (AUC) values and area under precision-recall curve (AUPR) values are computed for performance comparison.

Furthermore, comprehensive drug-disease association predictions using all known information as training set are conducted. We analyse the top-ranked results for the 548 drugs by searching evidence from public databases.

### Prediction performance comparison

**Fig. 1:**
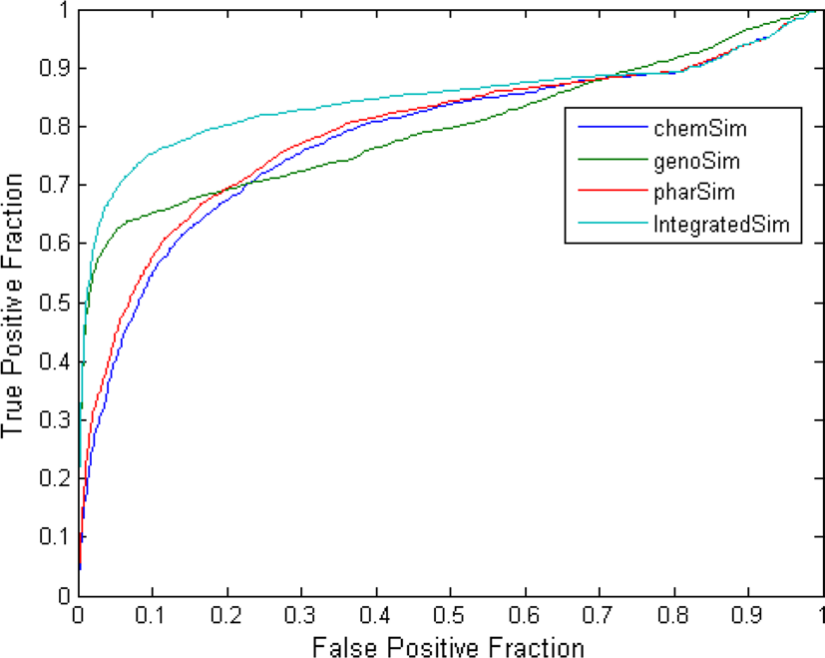
ROC curves of the 4 similarity measurements to predict drug-disease associations by leave-one-out cross-validation tests

**Fig. 2:**
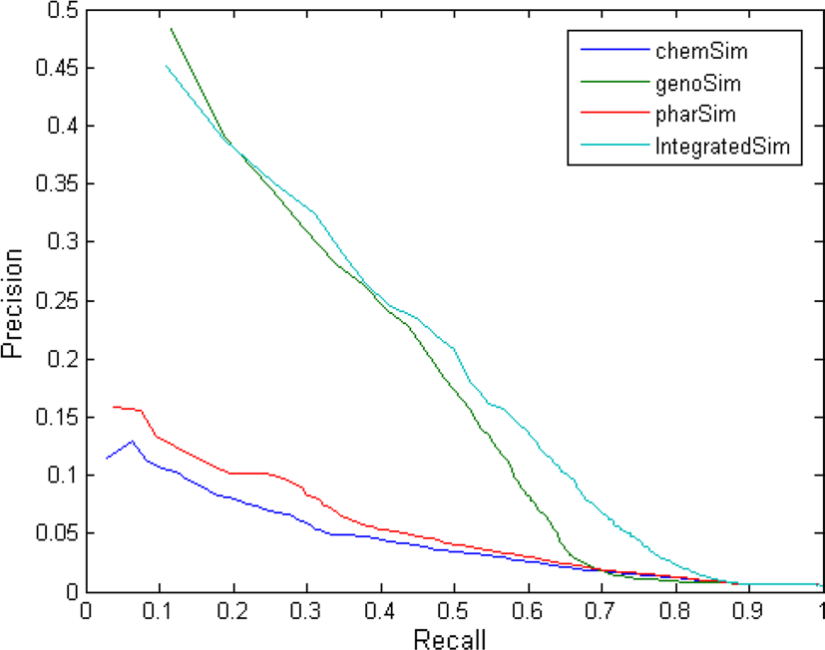
PR curves of the 4 similarity measurements to predict drug-disease associations by leave-one-out cross-validation tests

We report in Table 1 the average AUC values and AUPR values received by LOOCVs on the 548 drugs from the 4 similarity measurements. As shown in Table 1, *IntegratedSim* receives the highest average values of AUC and AUPR and performs best in the 4 similarity measurements. The average AUC value for *IntegratedSim* increases by 0.0659, 0.0310 and 0.0536 than these for the other 3 measurements, respectively. Meanwhile, the average AUPR value for *IntegratedSim* is 0.1474, 0.0586 and 0.1289 higher compared with these for the other 3 measurements, respectively. The overall results of LOOCVs for all 4 similarity measurements are illustrated by ROC curves and PR curves in Figs. 1 and 2, respectively.

**Table 1:** Comparison of average values of AUC and AUPR received for the 548 drugs in the 4 similarity datasets by leave-one-out cross-validations

	*chemSim*	*genoSim*	*pharSim*	*IntegratedSim*
Average AUC value	0.7792	0.8141	0.7915	**0.8451**
Average AUPR value	0.0727	0.1615	0.0912	**0.2201**

The bold value indicated the highest one in each row

We conduct paired *t*-tests to measure whether the AUC values and AUPR values obtained by *IntegratedSim* across the 548 drugs are significantly higher than these in the other 3 datasets. The calculated *p*-values are available at Table 2. We can discover from the statistical results that *IntegratedSim* achieves significantly better performance than all the other 3 measurements at the significance level 0.05.

**Table 2:** Pairwise comparison with paired *t*-tests on the performance results obtained by *IntegratedSim* and the other 3 measurements across the 548 drugs

	*chemSim*	*genoSim*	*pharSim*
*p*-value between *IntegratedSim* and another similarity measurement based on AUC values	3.0384E−06	0.02104148	0.00015592
*p*-value between *IntegratedSim* and another similarity measurement based on AUPR values	1.70373E−32	3.89275E−05	2.11912E−23

**Fig. 3:**
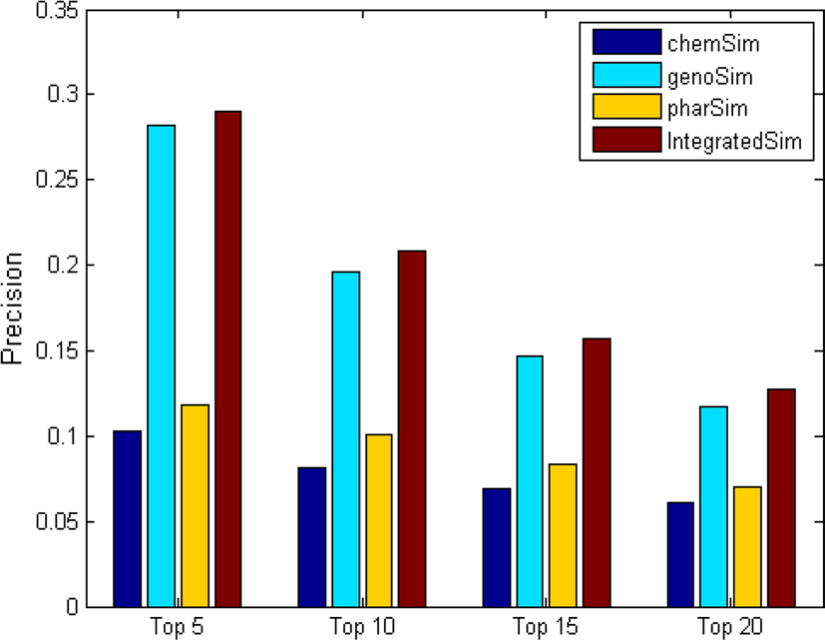
Comparison of average precision values in the top-*k* predictions for the 548 drugs in the 4 similarity datasets by leave-one-out cross-validations

**Fig. 4:**
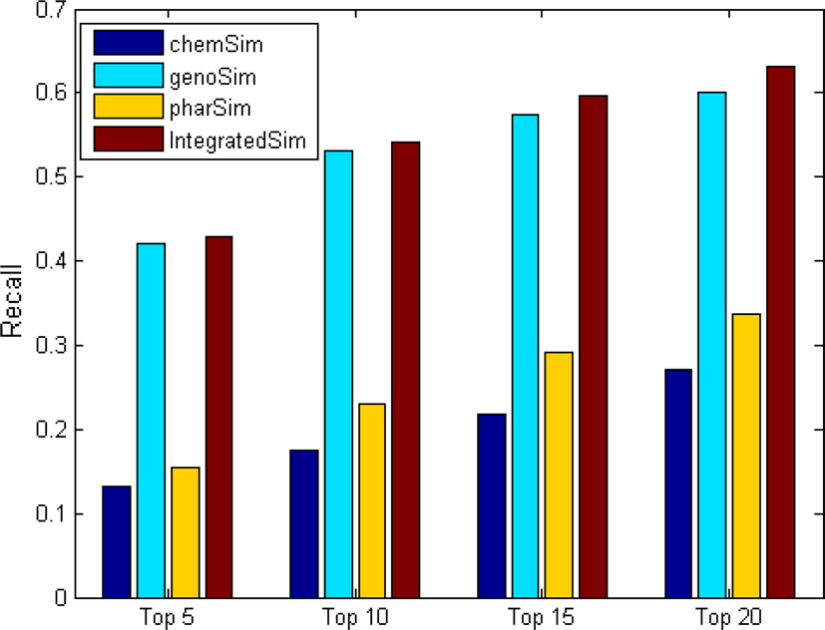
Comparison of average recall values in the top-*k* predictions for the 548 drugs in the 4 similarity datasets by leave-one-out cross-validations

We show the precision and recall values across the 548 drugs in the 4 similarity datasets within the top *k* (*k* = 5, 10, 15 and 20) candidates in Figs. 3 and 4, respectively. Because higher values of precision and recall within the top *k* predictions indicate that more real drug indications are successfully inferred. We can conclude from the two figures that *IntegratedSim* consistently outperforms the other 3 measurements at different *k* cutoffs.

### Effects of parameters *k* and *t* in similarity fusion on drug repositioning

There are two parameters *k* and *t* in our method for similarity fusion. The parameter *k* is the number of neighbours and *t* is the number of iterations. We comprehensively set their values in the range of [1, 30] and list the average AUC values and AUPR values in Tables 3 and 4, respectively. We find from the 2 tables that the best inference performance can be achieved when the values of both parameters are set to be 5.

**Table 3:** AUC values received from leave-one-out cross-validations by parameter tuning

	*k* = 1	5	10	20	30
*t* = 1	0.8313	0.8348	0.8287	0.8177	0.8100
5	0.8310	**0.8451**	0.8398	0.8320	0.8264
10	0.8307	0.8391	0.8273	0.8156	0.8096
20	0.8306	0.8253	0.8096	0.7989	0.7935
30	0.8306	0.8154	0.7993	0.7899	0.7862

The bold value indicated the highest one

**Table 4:** AUPR values received from leave-one-out cross-validations by parameter tuning

	*k* = 1	5	10	20	30
*t* = 1	0.2042	0.1868	0.1593	0.1342	0.1181
5	0.2084	**0.2201**	0.1943	0.1651	0.1537
10	0.2088	0.1962	0.1604	0.1357	0.1178
20	0.2085	0.1646	0.1296	0.0965	0.0835
30	0.2085	0.1456	0.1073	0.0822	0.0738

The bold value indicated the highest one

### Comprehensive prediction of novel drug–disease associations

After extensive comparison, we choose the best-performed similarity measurement *IntegratedSim* to conduct comprehensive drug-disease association predictions. In this inference process, all known information including associations and similarity measurement are used as the training set. We rank the unknown pairs according to their scores derived from Eq. (8). The list of the top 20 predicted results can be seen in Additional file 1.

**Fig. 5:**
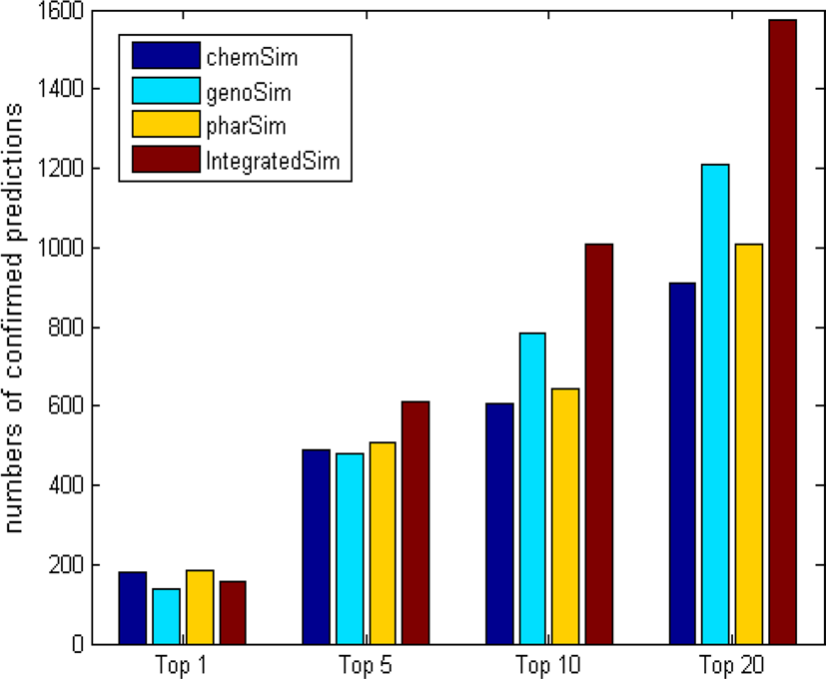
The numbers of confirmed results in the top-*k* predictions in the 4 similarity datasets

We check the top 20 predicted results according to the public database CTD [29], a knowledgebase that contains information for chemicals, genes, phenotypes, diseases, and exposures to advance our understanding about human health. Literature-based drug-disease associations are downloaded from this database to validate our predictions. For the predicted results in *IntegratedSim*, we discover that 158, 612, 1006 and 1575 predictions from the top 1, top 5, top 10 and top 20 results for the 548 drugs are verified in CTD, respectively. We also predict new drug-disease associations using the other 3 similarity measurements. Comparison of numbers of confirmed associations in the top *k* (*k* = 1, 5, 10 and 20) predictions is showed in Fig. 5. We receive the largest numbers of confirmed predictions from *IntegratedSim* in most cases. It should be noted that the top predictions that are not supported in CTD yet may also exist in reality.

### Comparison with other similarity fusion methods

We compare our integration method with 3 latest similarity fusion methods. We refer to the 3 methods as Napolitano’s method [30], Oerton’s method [31] and Li’s method [32]. To make fair comparison, we apply the 3 fusion methods to our datasets for drug-disease association prediction. We also use leave-one-out cross validations to test their prediction abilities. The average AUC and AUPR values of these methods are listed in Table 5. We discover that our method performs best in the 4 fusion methods.

**Table 5:** Comparison with 3 other similarity fusion methods based on leave-one-out cross-validations

	Napolitano’s method	Oerton’s method	Li’s method	Our method
Average AUC value	0.7989	0.7970	0.7993	**0.8451**
Average AUPR value	0.0974	0.0851	0.0986	**0.2201**

The bold value indicated the highest one in each row

## Discussion

Drug-based inference methods for drug repositioning make use of some unique drug features for matching. However, such information may be incomplete or contain noise. The incomplete or noisy data would produce biased results for drug repositioning.

We develop a method to combine 3 different drug features. We ensure in our integration method that a drug is more similar to itself than to other drugs throughout iterations, which results in more reliable drug-disease association predictions.

Note that the information of target proteins used in our manuscript is not complete. Meanwhile, according to a review [33], non-coding RNAs (ncRNAs) would be another new class of drug targets as they play significant roles in gene expression regulation and in disease progression. Integrating these ncRNAs with target proteins would make us know better about drug’s MoA. We therefore expect that the performance of our method would be improved when more experimentally supported drug targets are integrated.

In addition, our method could be easily extended when more drug features are available. This is useful because diverse categories of biomedical data are becoming available with recent advances in technologies. These biomedical data offer new potential for drug repositioning [34–37].

It should be noted that the performance of our similarity integration method depends on suitable parameter setting. Choosing proper parameters under different conditions for our method is a problem that needs to be properly addressed. Meanwhile, we only study the effects of drug features on drug repositioning. Recent repurposing approaches [38–41] are making using of both drug and disease data. Our previous study [42] demonstrated that the topology of drug-disease bipartite network is also a vital factor in predicting new indications for drugs. In the future, we plan to integrate more information to improve the prediction ability.

## Conclusions

In this paper, we comprehensively study the effects of 3 drug features from chemical, genomic and pharmacological spaces on drug repositioning. Cross-validations and case studies suggest the 3 drug features are all predictive factors for drug repositioning. We further develop a fusion method to integrate these features for better in silico drug repositioning. Compared with 3 latest state-of-the-art methods, our fusion method shows improvements in prediction accuracy. We expect that our study will provide guidance in data integration for in silico drug repositioning.

## Methods

### Data preparation

In our manuscript, we collect and integrate 3 types of drug signatures for drug repositioning. The datasets used for performance evaluation and new drug indication prediction are downloaded from two references [43, 44].

In reference [43], Zhang et al. collected chemical structures of 1103 drugs from PubChem [45]. They used 881-dimensional binary fingerprint profiles to encode the presence or absence of substructures. Target proteins of 1007 drugs were obtained from DrugBank [46]. Each drug was represented by a 775-dimensional binary target profile. Side-effects of 888 drugs were received from SIDER [47]. They used 1385-dimensional binary profiles to encode the presence or absence of each side-effect keyword.

In reference [44], Li and Lu extracted therapeutic uses for 799 drugs from NDF-RT (http://www.nlm.nih.gov/research/umls/sourcereleasedocs/current/NDFRT/) and provided 3250 drug-disease relationships between the 799 drugs and 719 diseases. Finally, we receive 548 drugs which contain all information of chemical structures, target proteins, side-effects and indications.

### Similarity calculation and fusion

As there are three types of drug features (chemical structures, target proteins and side-effects) in our study and these features are represented by binary profiles, we separately calculate the similarity between drugs in each feature set according to the Jaccard score. This strategy of similarity calculation is also applied in reference [48], in which the similarity score between two drugs based on the feature of chemical structures is computed as the size of the intersection over the union when viewing each chemical structure as specifying a set of elements. We refer to the 3 similarity datasets as *chemSim*, *genoSim* and *pharSim*.

Inspired by the successful work of reference [49] in shape/image retrieval and reference [50] in cancer subtype identification, we apply a diffusion method as follows to combine the 3 calculated similarity measurements. We refer to this integrated similarity as *IntegratedSim*.

For generality, we use an $n \times n$ similarity matrix $W$ with $W(i,j)$ indicating the similarity between drug $x_{i}$ and drug $x_{j}$. We define a full and sparse kernel on the similarity matrix $W$ and the full kernel is normalized as:

1 $$P(i,j) = \left\{ {\begin{array}{*{20}l} {{\raise0.7ex\hbox{${W(i,j)}$} \!\mathord{\left/ {\vphantom {{W(i,j)} {\left( {2\sum\nolimits_{k \ne i} {W(i,k)} } \right)}}}\right.\kern-\nulldelimiterspace} \!\lower0.7ex\hbox{${\left( {2\sum\nolimits_{k \ne i} {W(i,k)} } \right)}$}}} \hfill & {j \ne i} \hfill \\ {1/2} \hfill & {j = i} \hfill \\ \end{array} } \right.$$

Let $N_{i}$ represent a set of drug $x_{i}$’s neighbours. We use K nearest neighbours (KNN) to measure local affinity as:

2 $$S(i,j) = \left\{ {\begin{array}{*{20}l} {{\raise0.7ex\hbox{${W(i,j)}$} \!\mathord{\left/ {\vphantom {{W(i,j)} {\sum\nolimits_{{k \in N_{i} }} {W(i,k)} }}}\right.\kern-\nulldelimiterspace} \!\lower0.7ex\hbox{${\sum\nolimits_{{k \in N_{i} }} {W(i,k)} }$}}} \hfill & {j \in N_{i} } \hfill \\ 0 \hfill & {{\text{otherwise}}} \hfill \\ \end{array} } \right.$$

Suppose there are 2 similarity datasets for fusion. We compute $P^{(1)}$ and $P^{(2)}$ according to Eq. (1) for the two similarity matrices; then the matrices $S^{(1)}$ and $S^{(2)}$ are calculated as in Eq. (2). Let $P_{t = 0}^{(1)} = P^{(1)}$ and $P_{t = 0}^{(2)} = P^{(2)}$ denote the initial two status matrices when *t* = 0. We propagate the similarity information through the common neighbourhood and update the two similarity matrices iteratively as follows:

3 $$P_{t + 1}^{(1)} = S^{(1)} \times P_{t}^{(2)} \times (S^{(1)})^{T}$$

4 $$P_{t + 1}^{(2)} = S^{(2)} \times P_{t}^{(1)} \times (S^{(2)})^{T}$$

After *t* steps, the final integrated similarity matrix is computed as

5 $$Sim_{final} = \frac{{P_{t}^{(1)} + P_{t}^{(2)} }}{2}$$

For the 3 similarity measurements in our study, we adjust Eq. (3) to

6 $$P_{t + 1}^{(1)} = S^{(1)} \times \frac{{P_{t}^{(2)} + P_{t}^{(3)} }}{2} \times (S^{(1)} )^{T}$$

The final fused similarity matrix is calculated as

7 $$Sim_{final} = \frac{{P_{t}^{(1)} + P_{t}^{(2)} + P_{t}^{(3)} }}{3}$$

### Drug-disease association prediction

**Fig. 6:**
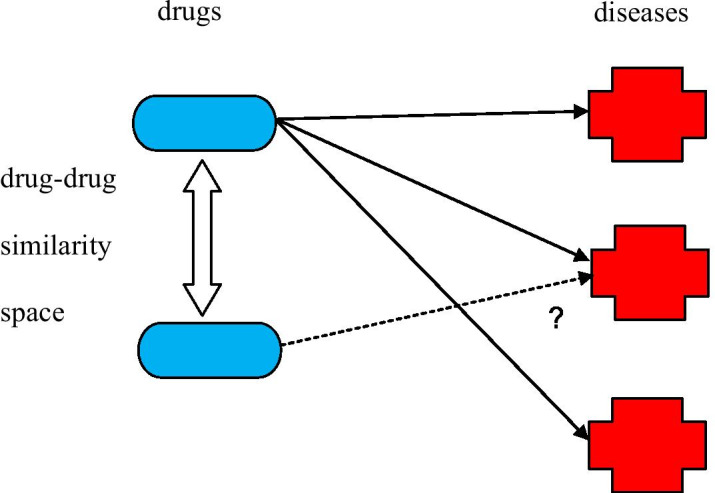
The guilt-by-association principle behind our in silico drug repositioning. If a drug with unknown indication profile shares a similar property with another drug whose indication profile is known, the former may share the same indication profile with the latter

Based on the guilt-by-association principle, we assume if a drug is prescribed to treat a disease, similar drugs might also be able to cure the disease (see Fig. 6). The same idea for association analysis has been used in some other bioinformatics fields [51–53].

For an unknown drug-disease association (*r*_*i*_, *d*_*j*_), we calculate its inference score as,

8 $$score(r_{i} ,d_{j} ) = \frac{{\sum\nolimits_{l = 1,l \ne i}^{n} {Sim(r_{i} ,r_{l} )a_{lj} } }}{{\sum\nolimits_{l = 1,l \ne i}^{n} {Sim(r_{i} ,r_{l} )} }}$$

where *r*_*i*_ and *d*_*j*_ denote drug *i* and disease *j*, $Sim(r_{i} ,r_{l} )$ is the similarity value between drugs *i* and *l*, and $a_{lj}$ = 1if there exists an association between drug *l* and disease *j*, otherwise $a_{lj}$ = 0. The higher a score is received from Eq. (8), the higher with confidence a prediction is. The top predicted diseases are considered as new indications for drugs of interest.

## Supplementary Information

**Additional file 1.** The top 20 predicted indications for the 548 drugs based on the similarity measurement *IntegratedSim*.

## Data Availability

The datasets used and/or analysed during the current study are available from public databases. The links for these databases are as follows. PubChem: https://pubchem.ncbi.nlm.nih.gov/ DrugBank: https://go.drugbank.com/ SIDER: http://sideeffects.embl.de/ CTD: http://ctdbase.org/

## Abbreviations

R&D: Research and development
SEA: Similarity ensemble approach
MoA: Mechanism of actions
KNN: K nearest neighbours
ncRNAs: non-coding RNAs
